# Single agent BMS-911543 Jak2 inhibitor has distinct inhibitory effects on STAT5 signaling in genetically engineered mice with pancreatic cancer

**DOI:** 10.18632/oncotarget.6332

**Published:** 2015-10-31

**Authors:** Thomas A. Mace, Reena Shakya, Omar Elnaggar, Kristin Wilson, Hannah M. Komar, Jennifer Yang, Jason R. Pitarresi, Gregory S. Young, Michael C. Ostrowski, Thomas Ludwig, Tanios Bekaii-Saab, Mark Bloomston, Gregory B. Lesinski

**Affiliations:** ^1^ Division of Medical Oncology, Department of Internal Medicine, The Arthur G. James Cancer Hospital and Richard J. Solove Research Institute, The Ohio State University, Columbus, OH 43210, USA; ^2^ Comprehensive Cancer Center, The Arthur G. James Cancer Hospital and Richard J. Solove Research Institute, The Ohio State University, Columbus, OH 43210, USA; ^3^ Veterinary Biosciences, The Arthur G. James Cancer Hospital and Richard J. Solove Research Institute, The Ohio State University, Columbus, OH 43210, USA; ^4^ Department of Molecular and Cellular Biochemistry, The Arthur G. James Cancer Hospital and Richard J. Solove Research Institute, The Ohio State University, Columbus, OH 43210, USA; ^5^ Center for Biostatistics, The Arthur G. James Cancer Hospital and Richard J. Solove Research Institute, The Ohio State University, Columbus, OH 43210, USA; ^6^ Division of Surgical Oncology, Department of Surgery, The Arthur G. James Cancer Hospital and Richard J. Solove Research Institute, The Ohio State University, Columbus, OH 43210, USA

**Keywords:** Jak2, STAT3, STAT5, pancreatic cancer

## Abstract

The Jak/STAT pathway is activated in human pancreatic ductal adenocarcinoma (PDAC) and cooperates with mutant *Kras* to drive initiation and progression of PDAC in murine models. We hypothesized that the small-molecule Jak2 inhibitor (BMS-911543) would elicit anti-tumor activity against PDAC and decrease immune suppressive features of the disease. We used an aggressive genetically engineered PDAC model with mutant *KrasG12D*, *tp53R270H*, and *Brca1* alleles (KPC-Brca1 mice). Mice with confirmed tumor burden were treated orally with vehicle or 30 mg/kg BMS-911543 daily for 14 days. Histologic analysis of pancreata from treated mice revealed fewer foci of adenocarcinoma and significantly decreased Ki67^+^ cells versus controls. *In vivo* administration of BMS-911543 significantly reduced pSTAT5 and FoxP3 positive cells within the pancreas, but did not alter STAT3 phosphorylation. Continuous dosing of KPC-Brca1 mice with BMS-911543 resulted in a median survival of 108 days, as compared to a median survival of 87 days in vehicle treated animals, a 23% increase (*p* = 0.055). *In vitro* experiments demonstrated that PDAC cell lines were poorly sensitive to BMS-911543, requiring high micromolar concentrations to achieve targeted inhibition of Jak/STAT signaling. Similarly, BMS-911543 had little *in vitro* effect on the viability of both murine and human PDAC-derived stellate cell lines. However, BMS-911543 potently inhibited phosphorylation of pSTAT3 and pSTAT5 at low micromolar doses in human PBMC and reduced *in vitro* differentiation of Foxp3^+^ T regulatory cells. These results indicate that single agent Jak2i deserves further study in preclinical models of PDAC and has distinct inhibitory effects on STAT5 mediated signaling.

## INTRODUCTION

Recent published models predict that pancreatic ductal adenocarcinoma (PDAC) will surpass breast and colon cancer, becoming the second leading cause of cancer-related deaths by the year 2030 [1]. Currently, PDAC is the 4^th^ leading cause of cancer related death in the world, and in the United States has a dismal 5-year survival rate of less than 7 percent [2]. One major difficulty with PDAC is its clinical silence. Typically the disease only becomes apparent after the tumor invades surrounding tissues or metastasizes to distant organs [3]. For many years, the current standard of care for most advanced PDAC patients has been gemcitabine. However slight improvements in overall survival are emerging with combination treatment using gemcitabine and nab-paclitaxel (Abraxane) [4], or aggressive chemotherapy regimens (e.g. FOLFIRNOX) as a strategy to de-bulk the tumor and improve candidacy for surgery [5]. Regardless, these advances may rightfully be classified as only incremental, and justify further research to identify novel strategies with potential for long term clinical responses and cures for this devastating malignancy.

The Janus kinases (JAK) are a family of tyrosine kinases that mediate signal transduction through the phosphorylation of signal transducer and activator of transcription (STAT) proteins, which regulate gene expression important for survival, proliferation, and differentiation. Recently, activation of the Interleukin-6 (IL-6)/Jak/STAT pathway has been shown to be associated with poor outcome and response to chemotherapy in PDAC patients [6, 7]. Furthermore, cytokine mediated signaling through the Jak/STAT pathway is a means by which immunosuppressive cell populations such as T regulatory cells expand in patients with advanced malignancy [8]. Indeed, these and other immune subsets including Th17 cells or myeloid derived suppressor cells (MDSC) are correlated with poor outcome in PDAC when present at high levels in either the tumor microenvironment or in circulation [9, 10].

Targeting the Jak/STAT signaling pathway in cancer is the focus of numerous pre-clinical and clinical studies due to its constitutive activation in many different tumors. Pre-clinical studies have shown efficacy of pan-Jak1/2 inhibition in many tumors such as myeloproliferative neoplasms [11], lymphoma [12], NSCLC [13], ovarian [14], and gastric cancer [15]. However, targeted therapies may act not only via the tumor but also alter signaling pathways involved in expansion of immune suppressive cell populations. Jak/STAT signaling is normally activated transiently in healthy immune cells however many factors (IL-6, IL-10, TGF-β, VEGF) secreted by pancreatic tumor or stromal cells could lead to constitutive Jak/STAT activation and subsequent differentiation of immunosuppressive populations (Treg, MDSC, Th17) [16–18]. Thus, examining these extrinsic effects on host immune modulation during Jak/STAT inhibition may be important in uncovering a mechanism of action and finding suitable targets for combination therapies.

This current pre-clinical study tested a single agent Jak2 inhibitor (BMS-911543; Bristol-Myers Squibb) in an aggressive genetically engineered mouse model (GEMM) of PDAC. We hypothesized that the functionally selective, small-molecule Jak2 inhibitor would elicit anti-tumor activity against PDAC and decrease immune suppressive features of the disease. Single agent BMS-911543 showed evidence of improved histologic features of disease in this highly aggressive PDAC GEMM. Interestingly treatment with BMS-911543 was also associated with reduced STAT5 phosphorylation and FoxP3^+^ cells in the tumor microenvironment. *In vitro* experiments indicated greater sensitivity of immune cells to BMS-911543 as compared to pancreatic tumor or stellate cells. This drug was also an effective means to limit cytokine-driven expansion of T regulatory cells *in vitro*. These results are the first to characterize the effects of the BMS-911543 Jak2 inhibitor in an aggressive PDAC model and assess its ability to elicit distinct inhibitory effects on STAT5 signaling, a pathway relevant to immunosuppressive T regulatory cells.

## RESULTS

### Genetically engineered mouse models (GEMM) of PDAC display activation of Jak/STAT signaling

**Figure 1:**
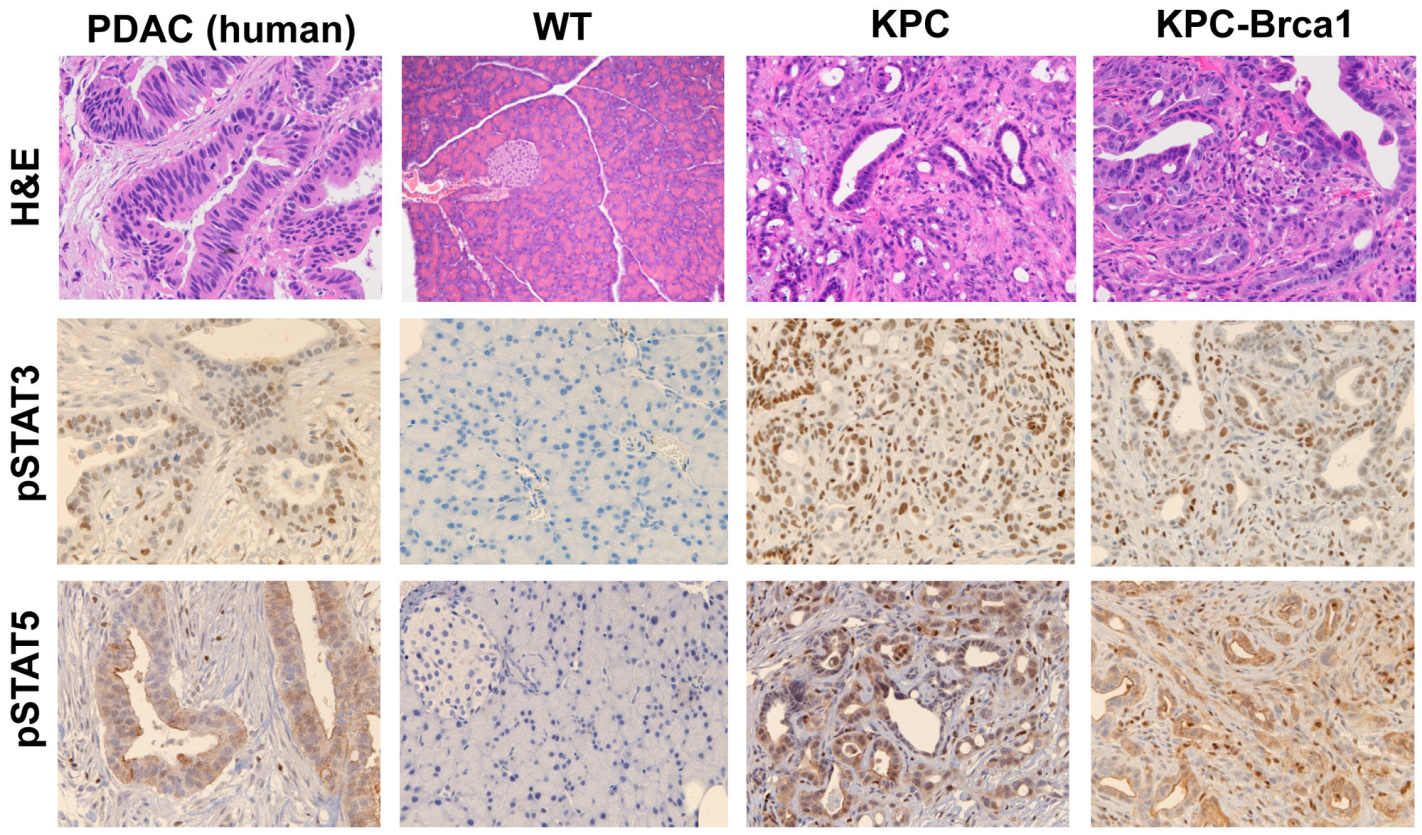
The Jak/STAT signaling pathway is active in genetically engineered mouse models (GEMM) of PDAC Representative 20x H&E, pSTAT3 (Tyr705), and STAT5 (Tyr694) immunohistochemistry images from pancreatic tissue from human PDAC, wild-type murine pancreas, KPC, and KPC-Brca1 mice.

Genetically engineered mouse models of pancreatic cancer can recapitulate many features of disease observed in patients. We evaluated activation of the Jak/STAT pathway in KPC mice and a highly aggressive variant of the KPC animals that concurrently incorporates the *Brca1* mutation. A majority of pancreatic cancer patients exhibit mutated *KRAS* and approximately 70% harbor *TP53* mutations [19]. *BRCA1/2* mutations are emerging as important genetic alterations in pancreatic cancer that are found in 5%–10% of patients [20, 21]. Compared to healthy wild type controls, high-grade PanIN and PDAC lesions were evident in GEMM such as KPC (mutant *KrasG12D*, *tp53R270H)* and KPC-Brca1 (mutant *KrasG12D*, *tp53R270H*, and *Brca1*) that resemble the pathology of pancreatic cancer patients (Figure 1). Interestingly, in KPC-Brca1 mice, neoplastic cyst formation was observed with the presence of papillary mucinous carcinoma similar to clinical observations in pancreatic cancer patients. Not only do these GEMM recapitulate human pancreatic cancer histologically, but they also have an active Jak/STAT signaling pathway, consistent with human tumors. We observed phosphorylation of STAT3 (Tyr 705) and STAT5 (Tyr694) in pancreatic tissue of KPC and KPC-Brca1 that localized to both the tumor and stromal cells. In contrast, few if any pSTAT3 or pSTAT5 positive cells were observed in pancreata from wild type control mice.

### *In vivo* administration of BMS-911543 limits biomarkers of tumor progression in KPC-Brca1 mice

**Figure 2:**
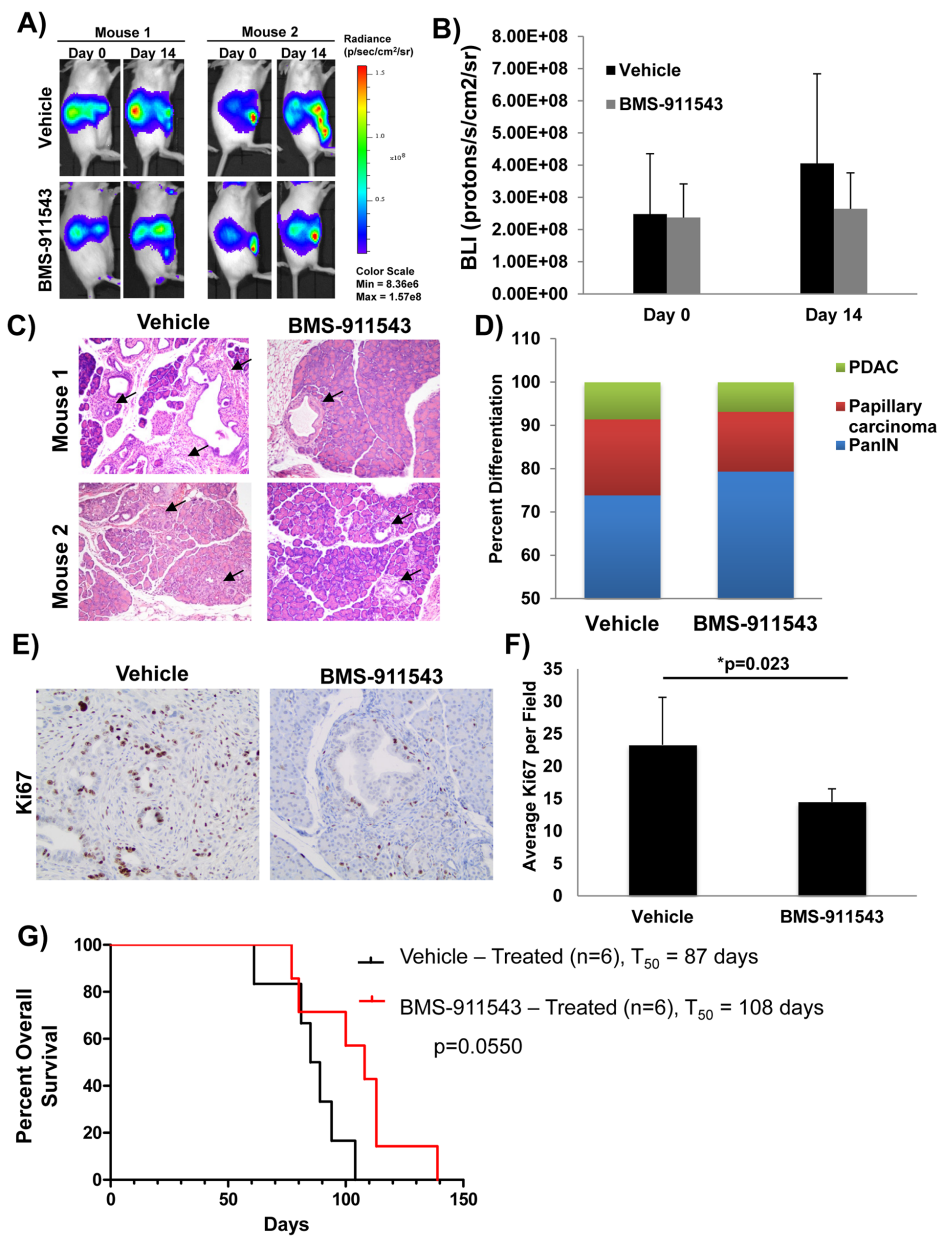
Effect of *in vivo* administration of BMS-911543 on biomarkers of tumor progression in the KPC-Brca1 GEMM Tumor burden of KPC-Brca1 mice was confirmed by **A.** bioluminescent (BLI) imaging at 5–6 weeks of age, at which point mice were treated with 30 mg/kg of BMS-911543 (*n* = 5) or vehicle (*n* = 5) by daily gavage for 14 days. **B.** BLI data were quantified in animals at this time point. **C.** H&E from pancreatic tissue obtained from two representative animals following euthanasia at day 14 post-treatment were analyzed and **D.** differentiation status was assessed in all specimens (*n* = 5). Arrows represent tumor burden assessed by histology. **E.** Representative IHC analysis of Ki67^+^ cells from day 14 tumors. **F.** Ki67^+^ cells were quantified in tumors from day 14 mice. **G.** At 8 weeks of age, KPC-Brca1 mice were treated with 30 mg/kg of BMS-911543 (*n* = 6) or vehicle (*n* = 6) by daily gavage until mice met pre-specified IACUC-approved early removal criteria.

To determine whether BMS-911543 elicits *in vivo* activity, the aggressive KPC-Brca1 GEMM was utilized. At 5–6 weeks of age, KPC-Brca1 mice (*n* = 5/group) were imaged by bioluminescent imaging (BLI) to confirm tumor burden, and then treated with vehicle or BMS-911543 Jak2i by oral gavage daily for 14 days (Figure 2A). BMS-911543 treated mice had an overall reduction in BLI signal as compared to the vehicle controls (Figure 2B). Consistent with BLI data, further histological analysis of pancreata from treated mice showed fewer foci of PDAC, with a shift toward PanIN lesions when compared to vehicle controls (Figure 2C–2D and Supplementary Figure S1). In addition, BMS-911543-treated mice had 35% fewer Ki67 positive cells as compared to vehicle treated animals (*p* = 0.023; Figure 2E–2F). The impact of BMS-911543 on overall survival was evaluated in KPC-Brca1 mice 8 weeks of age with more advanced tumors. Continuous dosing of KPC-Brca1 mice with single-agent BMS-911543 resulted in a modest (23%) increase in median survival of 108 days, as compared to a median survival of 87 days in vehicle treated animals, a 23% increase (log-rank *p* = 0.055; Figure 2G).

### BMS-911543 administration selectively decreases pSTAT5 in pancreatic tumors

**Figure 3:**
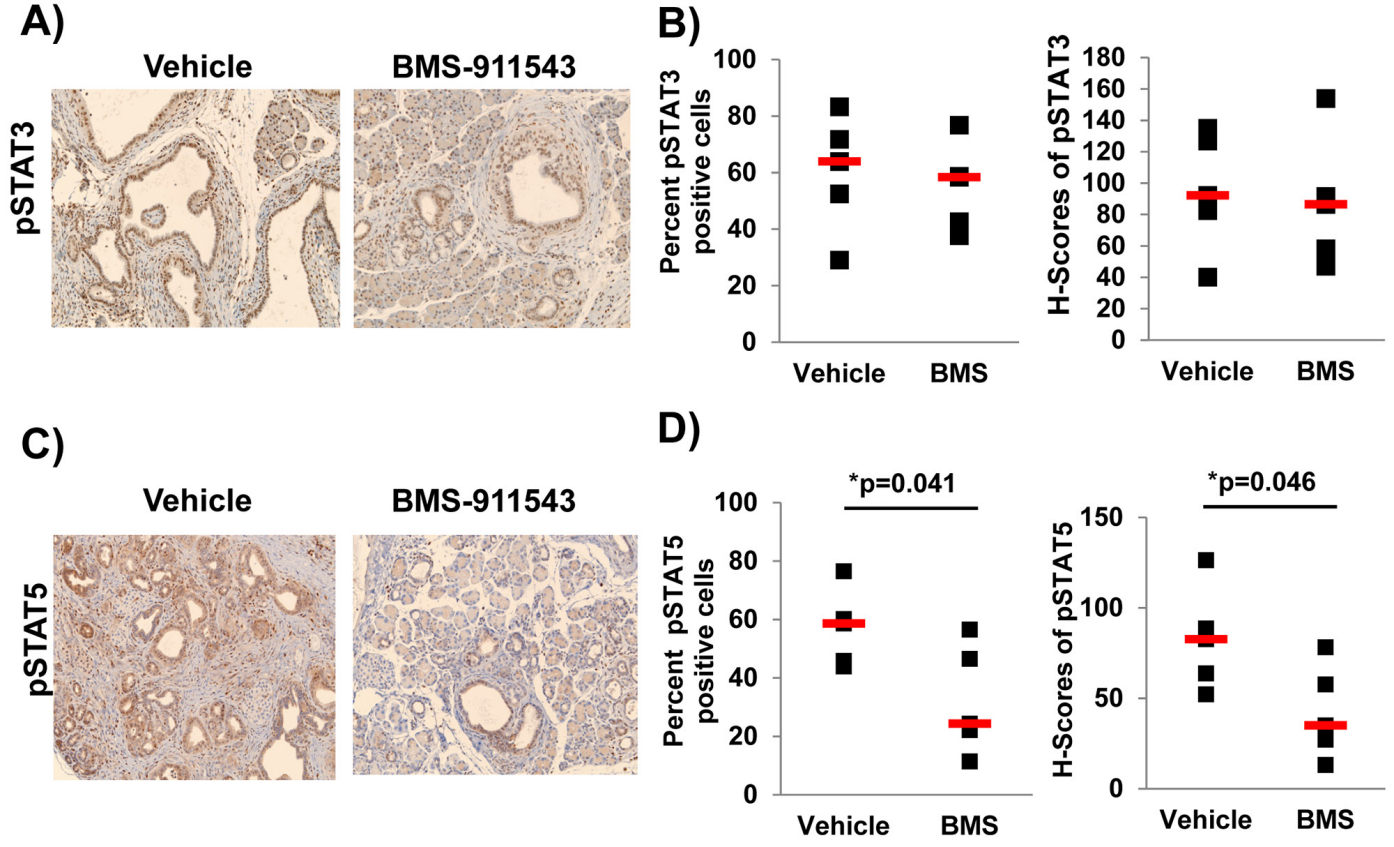
Administration of BMS-911543 decreases pSTAT5 expression PDAC tumors KPC-Brca1 mice (5–6 weeks of age confirmed by BLI were treated for 2 weeks with daily oral gavage of BMS-911543 at 30 mg/kg. **A** and **B.** pSTAT3 and **C** and **D.** pSTAT5 was assessed in the tissue by IHC (brown staining) at 20x magnification. (B) pSTAT3 and (D) pSTAT5 staining was quantified in the nucleus by spectral measuring of DAB intensity, which is represented as percent positive cells and H-score (staining intensity) from 5 random 20x fields per tissue/mouse (*n* = 5 per group).

To assess the impact of single agent BMS-911543 on Jak2 signaling *in vivo*, we utilized IHC to quantify phosphorylation of downstream Jak/STAT signaling intermediates including pSTAT3 (Figure 3A and 3B) and pSTAT5 (Figure 3C and 3D). BMS-911543 treatment did not significantly change either the percentage of pSTAT3 positive cells, or intensity of staining (H-score; determined using inform software tools; Supplementary Figure 2) in the pancreatic tissue (Figure 3B). In contrast, a significant decrease was observed in both the percentage of pSTAT5 positive cells (Figure 3D; *p* = 0.041) and the intensity of pSTAT5 staining (*p* = 0.046) in the pancreatic tissue of mice treated with BMS-911543 as compared to vehicle control (Figure 3D).

### Administration of BMS-911543 is associated with fewer intratumoral FoxP3^+^ T regulatory cells

**Figure 4:**
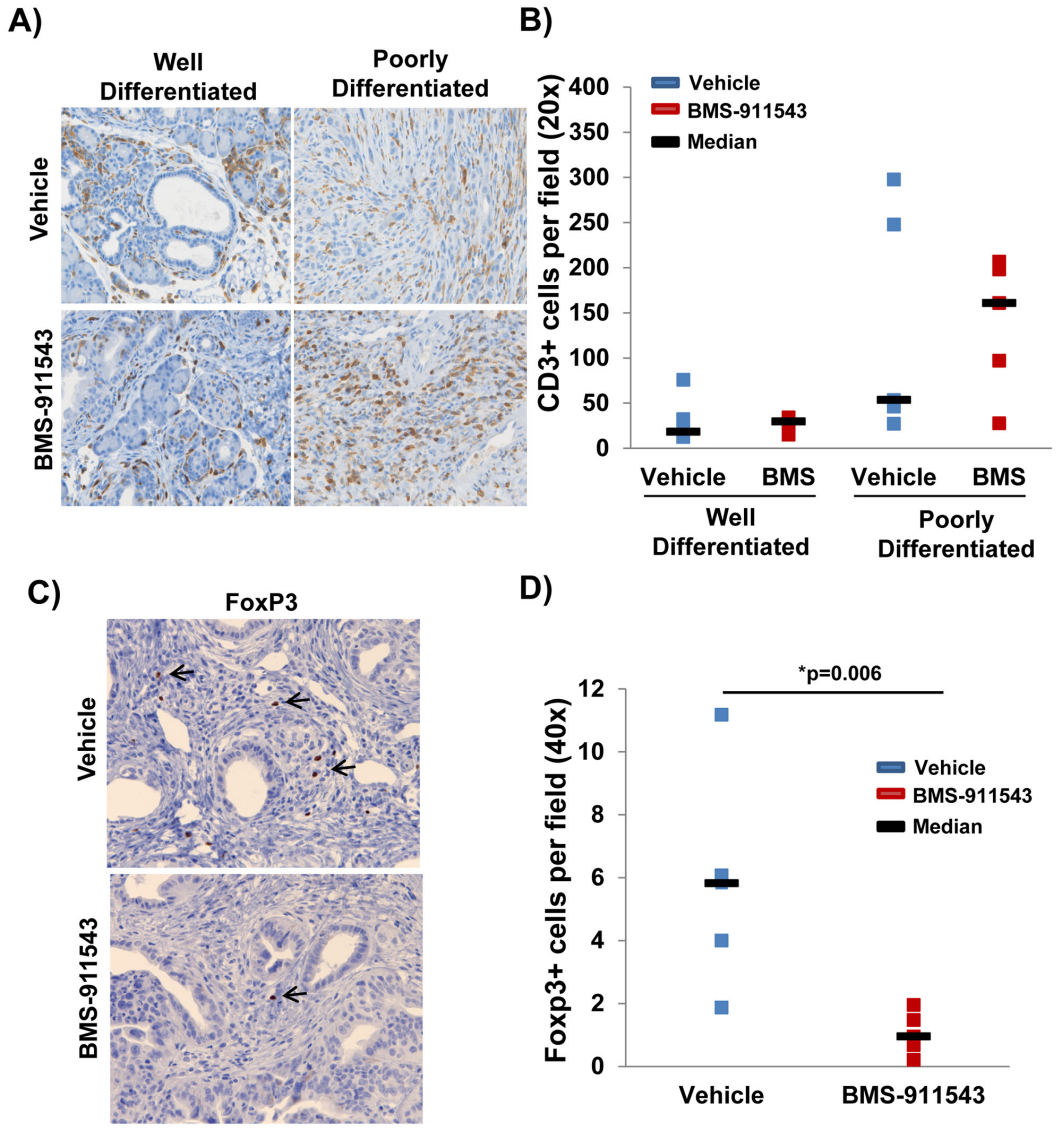
Reduced percentage of intratumoral FoxP3+ T regulatory cells in mice administered BMS-911543 KPC-Brca1 mice were treated with 30 mg/kg BMS-911543 for 2 weeks and pancreatic tissue was **A.** stained by IHC for CD3^+^ T cells (brown) in well-differentiated and poorly differentiated tumor areas and **B.** quantified by the average number of positive cells per field (40x magnification; *n* = 5 per group). **C.** Representative staining for FoxP3^+^ cells within the tumor of representative mice treated with either vehicle or BMS-911543. **D.** Quantification of FoxP3^+^ cells per field (40x magnification; *n* = 5 per group).

Histologic examination of the tumor microenvironment revealed a significantly greater proportion of CD3^+^ cells in poorly differentiated pancreatic tumor tissue as compared to well-differentiated tissue (*p* < 0.0001; Figure 4A and 4B). No difference in the total number of tumor infiltrating CD3^+^ T cells was observed between treatment groups in either well-differentiated or poorly differentiated tisse (*p* = 0.736; Figure 4B). However, further phenotypic analysis revealed a significant decrease in the number of FoxP3^+^ Tregs within the pancreata of BMS-911543 treated mice as compared to vehicle controls (*p* = 0.006; Figure 4C–4D). Bioplex analysis of plasma cytokines and chemokines from KPC-Brca1 mice following 2 weeks of treatment with BMS-911543 was also conducted to assess potential biomarkers of antitumor activity. A panel of cytokines and chemokines were assessed by multiplex analysis and no differences were observed in the plasma of mice treated with BMS-911543 compared to vehicle control (Supplementary Table S1). Phenotypic analysis of splenocytes revealed no difference in the percentage of different circulating immunosuppressive (granulocytic/monocytic MDSC, Tregs) and other immune subsets (DCs, B cells, T cells) between vehicle and BMS-911543 treated mice (Supplementary Table S2).

### *In vitro* sensitivity of pancreatic tumor, stromal, and immune effector cells to BMS-911543

**Figure 5:**
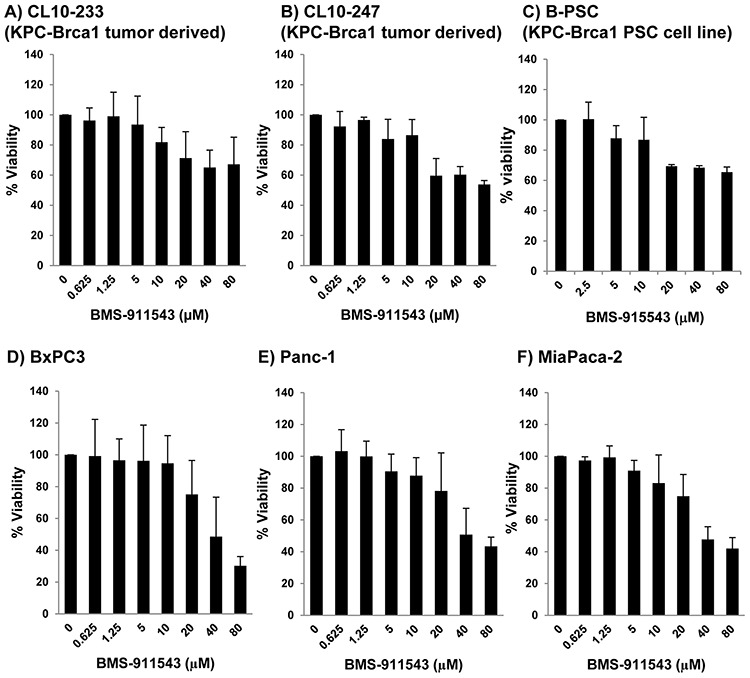
*In vitro* effects of BMS-911543 on murine and human PDAC and stellate cell lines **A** and **B.** Murine KPC-Brca1 PDAC, **C.** stellate cell lines, and human PDAC **D.** BxPC3, **E.** Panc-1, and **F.** MiaPaca-2 cell lines were treated *in vitro* for 48 hours with BMS-911543. Cell viability was measured by MTT assay. Error bars represent the standard deviation from 3 biological replicate experiments.

**Figure 6:**
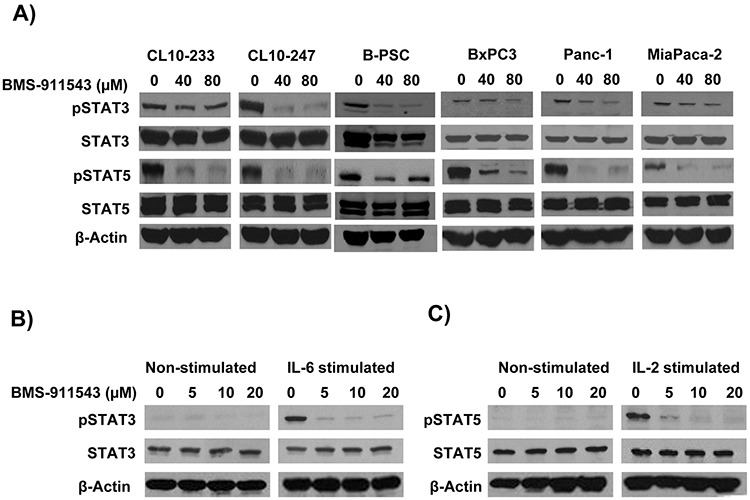
*In vitro* effects of BMS-911543 on cytokine-stimulated cells Cells were treated with BMS-911543 for 24 hours and lysates were made for western blotting. **A.** Murine KPC-Brca1 (CL10–233 and CL-243), B-PSC (KPC-Brca1 derived PSC line), and human PDAC (BxPC3, Panc-1, and MiaPaca-2) cell lines were analyzed for pSTAT3 and pSTAT5 by immunoblot. Healthy human donor PBMC were cultured for 2 hours with BMS-911543 and then stimulated for 20 minutes with **B.** recombinant IL-6 (10 ng/ml) to test for pSTAT3 or **C.** IL-2 (4 nM) to test for pSTAT5 expression. For all immunoblots β-actin was used as a loading control.

Our *in vivo* data suggest that Jak2 inhibition may elicit effects upon signaling within multiple cellular compartments. To further explore the relative sensitivity of individual cell compartments to BMS-911543, a series of *in vitro* studies was conducted. Murine and human PDAC cell lines were treated with increasing concentrations of BMS-911543 for 48 hours. Treatment of murine or human PDAC cell lines *in vitro* with BMS-911543 decreased proliferation only at concentrations greater than 20 μM. The absolute IC_50_ values for BMS-911543 were not achievable in any murine cell line (Figure 5A–5B), and were 39 μM or greater in all human PDAC cell lines tested (Figure 5D–5F). Primary SMA^+^ pancreatic stellate cells derived from murine KPC-Brca1 PDAC tissue were also treated *in vitro* with BMS-911543 (Figure 5C). Similar to PDAC cell lines, BMS-911543 had only modest effects on PSC proliferation, and did so only at high micromolar concentrations with an absolute IC_50_ value of 43.9. Immunoblot analysis of lysates from PDAC cell lines or PSC confirmed a limited inhibition of pSTAT3 which occurred only at higher concentrations of BMS-911543 (Figure 6A). In contrast, treatment of these cells with BMS-911543 potently inhibited STAT5 phosphorylation *in vitro*. The effect of BMS-911543 on Jak/STAT signaling was also evaluated in healthy donor PBMC. Because normal donors do not typically have basal phosphorylation of STAT proteins, these cells were cultured with BMS-911543 for 2 hours and then stimulated with 10 ng/ml IL-6 (to induce pSTAT3 signaling) or 4 nM IL-2 (to induce pSTAT5 signaling) for 20 min. In contrast to tumor and stellate cells, near complete inhibition of cytokine-induced STAT3 (Figure 6B) and STAT5 (Figure 6C) phosphorylation was observed after pre-treatment with BMS-911543. Of note, this inhibition of STAT signaling in healthy donor PBMC was evident at lower micromolar doses.

### BMS-911543 inhibits differentiation of T regulatory cells *in vitro*

**Figure 7:**
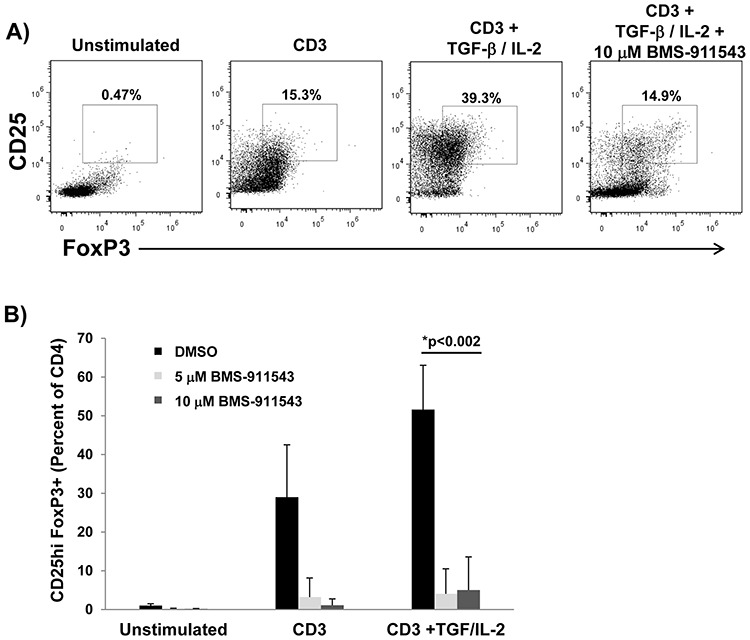
BMS-911543 inhibits T regulatory cell differentiation *in vitro* CD4^+^ T cells were negatively selected from healthy donor PBMC, treated with vehicle control or BMS-911543 (5 and 10 μM) and stimulated with 1 μg/ml CD3, 5 ng/ml IL-2, and 2 ng/ml TGF-β1 for 6 days. **A.** Cells were harvested and stained by flow cytometry by gating on CD4^+^CD25^hi^Foxp3^+^ cells. **B.** Data shown represent the mean percentage of CD25^hi^Foxp3^+^ cells as the percentage of CD4^+^ cells. Error bars represent the standard deviation of independent experiments using cells from *n* = 6 healthy donors.

Differentiation of T regulatory cells is mediated by STAT5 signaling. A series of *in vitro* studies were conducted to assess whether BMS-911543 could inhibit cytokine-mediated T reg expansion. For these studies, CD4^+^ T lymphocytes isolated from healthy normal PBMC were cultured with vehicle control or BMS-911543 and stimulated with plate-bound CD3, 5 ng/ml IL-2 and 2 ng/ml TGF-β1 for 6 days. In these experiments, BMS-911543 significantly inhibited *in vitro* expansion of CD4^+^CD25^hi^Foxp3^+^ T regulatory cells (Figure 7A–7B; *p* = 0.002).

## DISCUSSION

In the present study we demonstrate that Jak2 inhibition with BMS-911543 can alter histologic features of disease and limit STAT5 signaling in an aggressive model of PDAC. These results represent the first report of this targeted agent in pre-clinical models of PDAC. Our results provide preliminary data indicating that this agent may act at the level of STAT5-mediated signaling and expansion of T regulatory cells. We also observed no tissue toxicity or safety issues (no overt behavioral change or weight loss) with continuous treatment of mice with BMS-911543. These results are significant given the urgency for developing novel treatment approaches for an almost universally fatal disease.

The Jak/STAT pathway represents an attractive target in PDAC [22]. This pathway is constitutively active in human PDAC tumors and has been correlated with poor survival [6]. Clinical use of Jak inhibitors in pancreatic cancer has recently emerged, mostly in combination with chemotherapy. In a randomized double-blind phase 2 study, the Jak1/2 inhibitor, Ruxolitinib was combined with capecitabine and showed an improvement in overall survival as second-line therapy in patients with metastatic pancreatic cancer [23]. Several other pre-clinical studies have also examined the role of Jak inhibition in different murine pancreatic cancer models [24, 25]. For example, in an orthotopic xenograft model, Ruxolitinib elicited anti-tumor and anti-angiogenic effects *in vivo* [26]. Other *in vitro* studies have demonstrated that targeting the Jak/STAT pathway elicits apoptosis in PDAC cell lines [25, 27, 28]. Our pre-clinical work further builds upon these observations to characterize the activity of the BMS-911543 Jak2 inhibitor in a novel and highly aggressive murine model of spontaneously arising pancreatic cancer.

Distinct from these prior reports using Jak2 inhibitors in PDAC, our data suggest that STAT5 signaling may be a target of BMS-911543. Both *in vitro* and *in vivo* data support this mechanism, and preliminarily suggest this may act in part, at the level of T regulatory cells. Indeed, this transcription factor is instrumental in regulating the IL-2 mediated survival and expansion of T regulatory cells which is consistent with observations in this animal model [29]. This potential immunomodulatory effect deserves further investigation in additional mechanistic studies. In contrast, a more comprehensive analysis of cytokines, chemokines and cellular biomarkers indicated few if any systemic changes were evident upon analysis of splenocytes from these animals. These data indicate the importance of examining the effect of targeted agents on the tumor microenvironment.

Despite the anti-tumor effect observed in an aggressive murine PDAC model, there are a number of considerations that deserve mention when interpreting data from this study. First, single agent Jak2 inhibition provided evidence of benefit but was not curative. This observation emphasizes the need to understand mechanisms of resistance to single agent Jak inhibitors and to pursue combined therapy approaches in future pre-clinical animal studies. Second, although a consistent effect was observed on pSTAT5, little effect of BMS-911543 was evident on pSTAT3. This was consistent for both *in vitro* and *in vivo* studies. This was not entirely surprising due to prior reports of resistance to Jak2 inhibition mediated by constitutive STAT3 phosphorylation [30].

In conclusion, our studies characterize the activity of Jak2 inhibition using BMS-911543 in an aggressive pre-clinical model of PDAC. *In vitro* experiments provided additional evidence suggesting that immune cells may be more sensitive at the level of signal transduction, as compared to pancreatic tumor or stellate cells. Further mechanistic studies will be informative for understanding the relative contribution of limiting STAT5 signaling in the tumor and immune compartments as mediators of drug activity. These data are encouraging in the context of pre-clinical studies that build off of Jak2 inhibitors as a part of combined therapy regimens for PDAC.

## MATERIALS AND METHODS

### Cell lines and reagents

Human PANC-1, MiaPaca-2, BxPC-3 pancreatic cancer cell lines were cultured in RPMI or DMEM (Gibco) with 10% FBS, 10 mM L-glutamine, and antibiotics. Murine pancreatic cancer cell lines (CL10–233 and CL10–247) were derived from KPC-Brca1 mice. BMS-911543 (Jak2i inhibitor) was obtained from Bristol Meyers Squibb (BMS; New Jersey). As previously reported, BMS-911543 (N,N-dicyclopropyl-4-((1,5-dimethyl-1H-pyrazol-3-yl)amino)-6-ethyl-1-methyl-1,6-dihydroimidazo[4,5-d]pyrrolo[2,3b]pyridine-7-carboxamide) was prepared in dimethylsulfoxide for *in vitro* experiments or in 20% citrate/80% PEG400 vehicle for *in vivo* experiments [31]. Recombinant human IL-6, was purchased from Peprotech, Inc. (Rocky Hill, NJ) and recombinant human IL-2 and TGF-β1 were purchased from R&D Systems (Minneapolis, MN). MTT reagent was purchased from ATCC (Manassas, VA). Annexin V and propidium iodide were purchased from BD Biosciences (San Jose, CA).

### Genetically engineered mouse models of pancreatic cancer

The Brca1-KPC (*Brca1^flox2/flox2^; Kras^LSL-G12D/+^; p53^LSL-R270H/+^; Pdx1-cre*) mice have been previously described [32]. In brief, Brca1-KPC mice were generated by interbreeding *Brca1^flox2/flox2^; Kras^LSL-G12D/+^ with Brca1^flox2/flox2^; p53^LSL-R270H/+^; Pdx1-cre* animals. The *Brca1^flox2^* mice have been previously described [33]. The mouse strains *p53^LSL-R270H^* (strain number 01XM3), *Kras^LSL-G12D^* (strain number 01XJ6), and *Pdx1-cre* (strain number 01XL5) were acquired from the National Cancer Institute (NCI) Frederick Mouse Repository. Due to the number of alleles, it was necessary to maintain mice on a mixed 129/B6 genetic background. To allow quantitative bioluminescent imaging, KPC-Brca1 mice were crossed to have white fur, and express the luciferase transgene. A mCherry-luciferase 2 (mCL) fusion gene cassette was amplified by PCR to introduce *Sal*I and *Not*I sites at the 5′ and 3′ ends, respectively. A *Spe*I-*Sal*I fragment containing a *lox*P-flanked promoterless neomycin expression cassette (*lox*P-neomycin-3 copies of the simian virus 40 polyadenylation signal (SV40polyA)-*lox*P) and the *Sal*I-*Not*I mCL fragment were inserted together into a *Spe*I-*Not*I digested pBigT vector [34]. The insert of the resulting plasmid, consisting of the adenoviral splice acceptor sequence followed by the *lox*P-flanked neomycin expression cassette (LNL), mCL and a SV40polyA cassette, was excised by *Pac*I-*Asc*I and inserted into the ROSA26-PA vector [34]. The final knock-in vector Rosa26^LNL-mCL^ was linearized with *SnaBI* and electroporated into 129/SV embryonic stem cells. G418-resistant ES clones were analyzed by Southern blotting and correctly targeted clones were injected into C57BL/6J blastocysts to generate germ-line transmitting chimeras. For use in *in vivo* imaging, *Rosa26^LNL-mCL/+^* mice were first backcrossed for three generations to C57BL/6-cBrd/cBrd/Cr mice (C57BL/6 albino; NCI, Frederick) to eliminate the light attenuation caused by dark (black and agouti) skin and fur and then mated to Brca1-KPC mice. These animals were interbred with a mouse strain carrying the Rosa26LSL-mCL allele. The Rosa26LSL-mCL allele is a conditional knock-in of the mcherry-luciferase transgene into the Rosa26 locus; the design of the conditional targeting construct consists of the mcherry-luciferase transgene being immediately preceded by a LoxP flanked stop codon, which allows tissue-specific and temporal control over the expression of the mcherry-luciferase fusion protein. This conditional knock-in strategy ensures that only cells that express cre-recombinase and have undergone cre-mediated recombination, such as Pdx1-cre expressing pancreatic tumor cells among the KPC animals, will express mcherry-luciferase protein which can be visualized either by fluorescence and/or BLI.

### *In vivo* treatment using BMS-911543

Pancreatic tumors were confirmed in KPC-Brca1 mice by bioluminescent imaging (BLI) at 5–6 weeks of age. Briefly, mice were be maintained on isofluorane anesthesia and imaged 10–15 minutes following intraperitoneal injection of Luciferin on a heated platform of a Xenogen Bioluminescence station in the Small Animal Imaging Core Laboratory, within our Comprehensive Cancer Center. Animals with a pancreatic mass of approximately 50–100 mm^3^ were randomized, and treatment was initiated the day following imaging. Mice were then treated for 2 weeks by daily oral gavage at a dose of 30 mg/kg BMS-911543. Following 2 weeks of treatment, animals were euthanized via CO_2_ asphyxiation followed by cardiac puncture. Plasma, splenocytes and tumor tissue were collected for further analysis. Pathology was assessed by H&E to determine differentiation state of the tissue as PanIN, papillary carcincoma or PDAC. PanIN was defined as epithelial lesions with basement membrane and were composed of epithelium with nuclear and cellular atypia that had papillary, micropapillary and/or pseudostratified architecture. Papillary carcinoma was defined as epithelial lesions which were incompletely confined by the basement membrane and formed papillary structures that projected into the lumen. PDAC was defined as epithelial lesions that formed large lobules of cells that were locally invasive and typically lacked tubular architecture with high degree of nuclear and cellular atypia present [35]. For long term *in vivo* experiments, 8 week old KPC-Brca1 mice with advanced disease were continuously treated by oral gavage at 30 mg/kg of BMS-911543 until mice met specified early removal criteria in IACUC approved protocols.

### Pancreatic stellate cell isolation and culture

Fresh tissue from KPC-Brca1 pancreatic tumors was dissected with a scalpel into 0.5–1 mm^3^ pieces, further dissociated using collagenase type II (Worthington Biochemical, Lakewood NJ), filtered using a 70 μM filter and then plated in 6-well 10 cm^2^ uncoated culture wells in DMEM with 10% FBS and antibiotics and incubated at 37°C. PSC typically grew out of the tissue in 2–3 weeks and were characterized by morphology and histological analysis of alpha-smooth muscle actin (SMA^+^) staining. PSC were maintained in culture with fresh media added twice weekly.

### Immunohistochemical (IHC) analysis

Formalin fixed pancreatic tissue from *in vivo* experiments was subjected to IHC analysis following staining with antibodies against pSTAT3 (Catalog 4904; Cell Signaling), pSTAT5 (Catalog ab30648; Abcam, Cambridge MA), and FoxP3 (Catalog 88–8111-40; ebioscience, San Diego, CA). For pSTAT3 and pSTAT5 analysis, 20x magnification images of pancreata (5 images per mouse) were quantified using PerkinElmer's Vectra multispectral slide analysis system. inForm software tools were used to segment sections on a cellular basis into cytoplasmic, nuclear, and membrane fractions. DAB staining was measured in each cellular compartment and quantified by the percentage of cells and H-score. H-score accounts for both spectral intensity and percentage of positive cells. For FoxP3 analysis, blinded histological analysis of staining in the pancreas was counted at 40x magnification, with at least 15 fields counted per mouse.

### Flow cytometry

Immunophenotypic analyses of splenoctyes from animals were assessed by flow cytometry. Antibodies to stain for MDSC were CD11b-APC (Clone M1/70; BD Biosciences), Ly6G-FITC (Clone 1A8; BD Biosciences), Ly6C-PE (Clone AL-21; BD Biosciences); for dendritic cells were CD11c (Clone HL3; BD Biosciences); for B cells B220-APC (Clone RA3–6B2; BD Biosciences), CD3-FITC (Clone 145–1011; BD Biosciences). T regulatory cells with a phenotype of CD4^+^CD25^+^FoxP3^+^ were evaluated using a commercially available kit (eBiosciences, San Diego, CA). For T cell activation markers, cells were stained with antibodies specific for CD4-PE-Cy7 (Clone RM4–5; BD Biosciences), CD8-PE-Cy7 (Clone 53–6.7; BD Biosciences), CD62L-PE (Clone MEL-14; BD Biosciences), and CD44-Bv650 (Clone IM7; Biolegend). To determine Th1 and Th2 phenotypes, cells were stained using fluorochrome conjugated antibodies targeted CXCR3-PE-Cy7 (Clone CXCR13–173; Biolegend), CCR4-PE (Clone 2G12; Biolegend), and CCR6-APC (Clone CK4-L3; BD Biosciences). Cells were incubated on ice for 30 minutes, washed, and fixed in PBS containing 1% formalin for flow cytometric analysis on a LSRII flow cytometer (BD Biosciences).

### MTT assay

Human and murine PDAC tumor cells or PSC were cultured in 96 well plates and the following day treated with BMS-911543 or DMSO vehicle control for 48 hours. After 48 hours, MTT reagent (ATCC) was added for 2 hours at 37°C. Samples were analyzed on a plate reader testing for absorbance at 450 nM.

### Immunoblot analysis

Cell lysates were assayed for protein expression by immunoblot analysis with antibodies (Ab) against STAT3 (Catalog 4904), pSTAT3 (Catalog 9145; Tyr705), STAT5 (Catalog 9363), pSTAT5 (Catalog 9351; Tyr694), and β-actin (Catalog 4967; Cell Signaling Technology, Danvers, MA). Following incubation with appropriate horseradish-peroxidase-conjugated secondary Ab, immune complexes were detected using the SuperSignal West Pico Chemiluminescent Substrate (Thermo Fisher Scientific).

### Peripheral blood mononuclear cell (PBMC) isolation

Peripheral blood mononuclear cells (PBMCs) were isolated from source leukocytes of healthy donors (American Red Cross, Columbus, OH) via density gradient centrifugation using Ficoll-Paque (Amersham, Pharmacia Biotech, Bjorkgatan, Sweeden) as described [10]. PBMCs from healthy donors from the American Red Cross (Columbus, OH) were cultured in 10% FBS, 10 mM L-glutamine, and 100 μg/ml penicillin/streptomycin in RPMI 1640 (Gibco).

### *In vitro* differentiation of T regulatory cells

T regulatory cells were differentiated *in vitro* as previously described [36]. Briefly, CD4^+^ T cells were negatively selected from source leukocytes (American Red Cross, Columbus, OH) using a rosette separation kit (STEMCELL Technologies; Vancouver, BC) and stimulated with 1 μg/ml plate-bound CD3 antibody, 5 ng/ml IL-2 and 2 ng/ml TGF-β1 for 6 days. Cells were harvested and stained by flow cytometry for canonical Treg markers CD4-APC (Catalog IMQ468U; Beckman Coulter), CD25-FITC (Clone M-A251; BD Biosciences), and Foxp3 (Clone 259D/C7; BD Biosciences).

### Statistics

Two-sample *t*-tests were used to compare IHC outcomes between the control and BMS-911543 treated mice. If necessary to meet the assumptions of constant variance and normality, outcomes were log-transformed. Kaplan-Meier methods and the log-rank test were used to compare overall survival between the two treatment groups. The 4-parameter logistic Hill model [37] was the assumed dose-response relationship for the cell viability experiments. Nonlinear least squares regression was used to estimate the model parameters and generate 95% confidence intervals for the IC50 estimates. A mixed-effects model was used to analyze the number of CD3^+^ cells between the well and poorly differentiated tissue samples. A random effect for mouse was included to account for the two samples per mouse and fixed effects for treatment, differentiation category and the interaction of the two. For the bioplex analysis of cytokines and chemokines, geometric means were calculated by treatment group as well as 95% confidence intervals for the fold differences. Paired *t*-tests were used to test the change in the proportions of CD4 in the T regulatory donor cell experiments. All analyses were performed using SAS v9.4 (SAS Institute, Cary, NC).

## SUPPLEMENTARY FIGURES AND TABLES



## Abbreviations

PDAC: pancreatic ductal adenocarcinoma
GEMM: genetically engineered mouse model
JAK: Janus kinases
STAT: signal transducer and activator of transcription
PanIN: pancreatic intraepithelial lesions
BMS: Bristol-Myers Squibb
Treg: T regulatory
MDSC: myeloid derived suppressor cell
