# Supplementary material for: Single agent BMS-911543 Jak2 inhibitor has distinct inhibitory effects on STAT5 signaling in genetically engineered mice with pancreatic cancer
Source: Oncotarget. 2015 Oct 31;6(42):44509–22. doi: 10.18632/oncotarget.6332 (PMC4792572; doi:10.18632/oncotarget.6332)
Supplement: Supplementary file 1 [file oncotarget-06-44509-s001.pdf]

SUPPLEMENTARY FIGURES AND TABLES

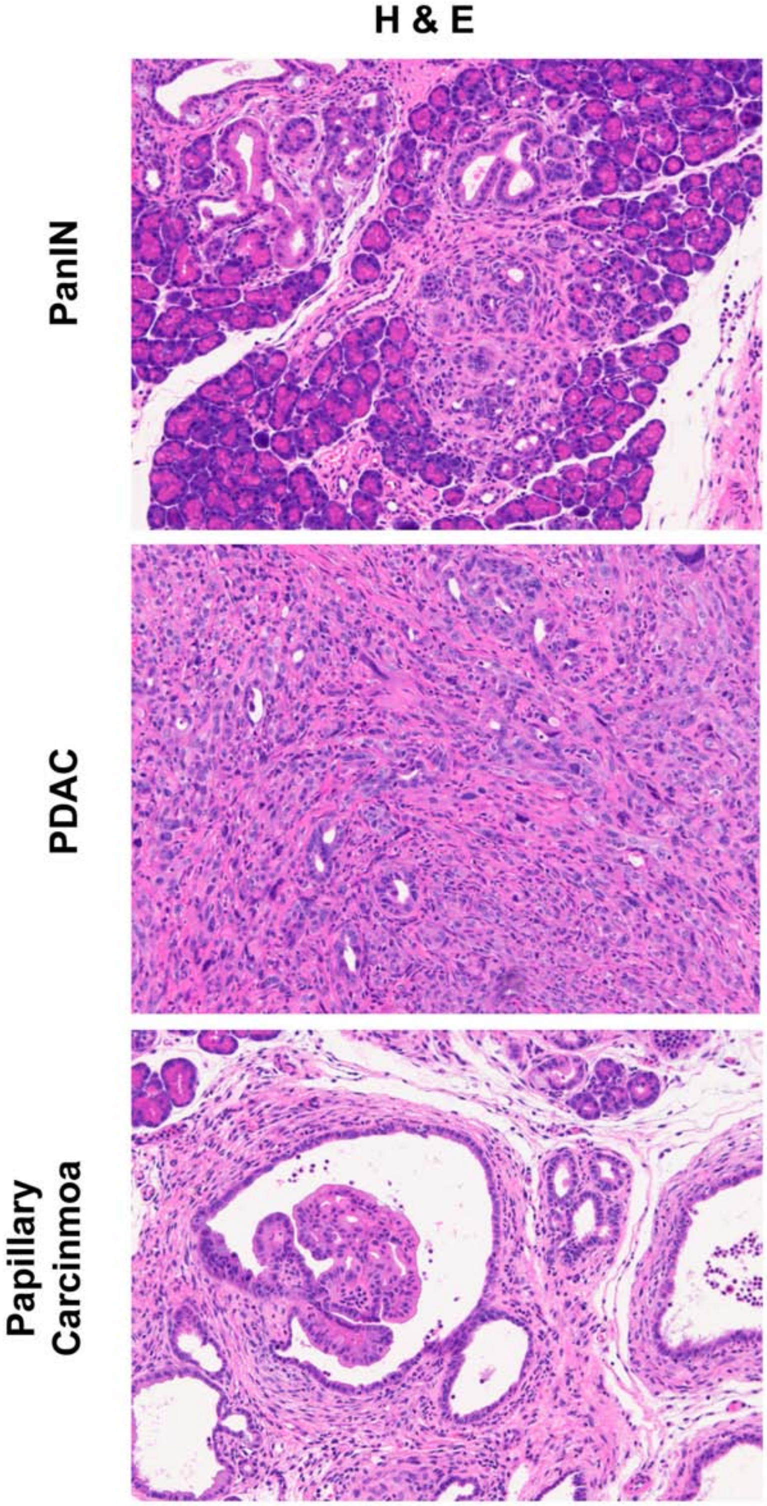

Supplementary Figure S1: Representative images of pathological features assessed in the pancreas of KPC-Brca1 mice. Pathology was assessed by H&E to determine differentiation state of the tissue. Representative 20x H&E images from pancreatic tissue of KPC-Brca1 described as PanIN, papillary carcincoma or PDAC.

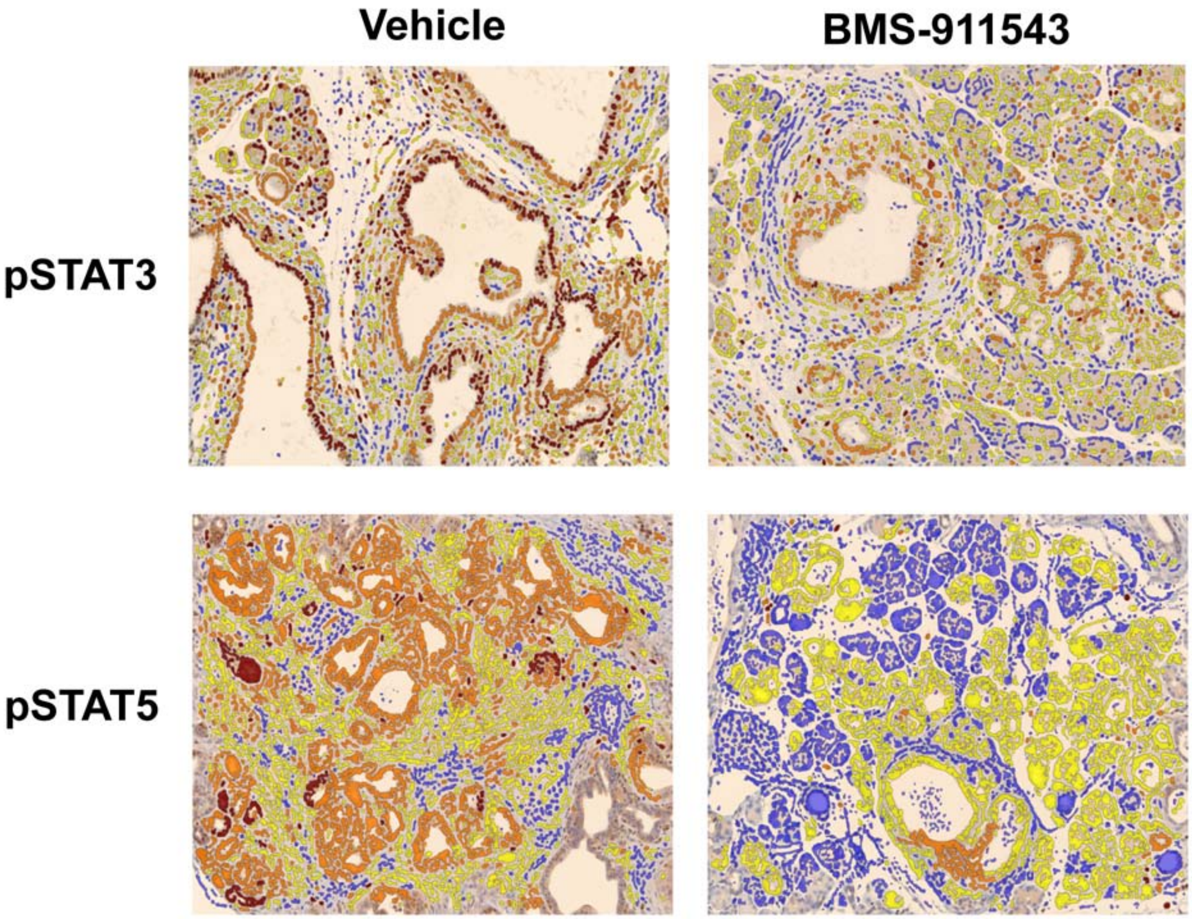

Supplementary Figure S2: Quantification of histological analysis of pSTAT3 and pSTAT5 in the pancreas of KPC-Brca1 mice. KPC-Brca1 mice (with confirmed tumor-burden by BLI) were treated daily by oral gavauge with 30 mg/kg BMS-911543 or vehicle control for 2 weeks. Paraffin embedded tumor tissue was stained for pSTAT3 and pSTAT5 in the pancreas and analyzed using PerkinElmer's Vectra multispectral slide analysis system and inForma software tools. Representative quantification images from Figure 3. Blue = negative, Yellow = more positive, orange = Very intense staining, and brown = highest intensity staining.

Supplementary Table S1: Cytokines and Chemokines from the plasma of BMS-911543 treated KPC-Brca1 mice

Cytokine	Vehicle geometric mean	BMS-911543 geometric mean	Fold difference (95% CI)
CCL11	269.4	783.1	2.91 (1.44, 5.85)
CXCL1	9.7	74.7	2.91 (1.44, 5.85)
CXCL5	3458.4	4553	1.32 (0.57, 3.06)
G-CSF	6.6	30.8	4.65 (0.41, 52.54)
IL-12p40	33.7	11.3	0.34 (0.03, 4.19)
IL-18	79.1	812.4	10.27 (0.51, 206.6)
IL-1beta	8.1	3.7	0.46 (0.08, 2.73)
IL-22	29.9	303.6	10.14 (0.25, 405.0)
IL-23	16.4	71	4.34 (0.04, 461.2)
IL-6	5.7	115.1	20.11 (1.14, 355.3)
IP-10	39.2	142.7	3.64 (0.78, 16.88)
MCP-1	9.7	13	1.34 (0.05, 34.86)
TNF-alpha	27.1	22.3	0.82 (0.36, 1.89)
VEGF	9.2	11.6	1.25 (1.09, 1.43)

KPC-Brca1 mice (with confirmed tumor-burden by BLI) were treated daily by oral gavage with 30 mg/kg BMS-911543 or vehicle control for 2 weeks. Plasma from mice was assessed for different cytokines and chemokines by bioplex analysis.

Supplementary Table S2: Immune subsets in the spleen of BMS-911543 treated KPC-Brca1 mice

	Vehicle		BMS-911543	
	Mean	STD	Mean	STD
Granulocytic (CD11b+Ly6G+Ly6C low)	1.17	0.49	2.58	1.55
Monocytic (CD11b+Ly6Gneg Ly6C High)	7.61	3.53	8.43	5.84
T-regulatory cells (CD4+CD25+FoxP3+)	2.93	0.56	2.26	0.76
Dentritic Cells (CD11c+ CD11b+)	8.95	6.98	6.33	3.80
B cells (B220 CD3-)	6.68	0.81	5.66	1.18
CD4				
CD62L+ CD44-	44.70	17.10	52.86	7.03
CD62L+ CD44+	28.14	4.38	25.80	5.10
CD62L- CD44+	11.35	9.59	9.42	3.86
CD62L- CD44-	10.06	4.29	7.13	2.71
Th1 (CXCR3+ CCR4- CCR6-)	18.24	4.84	17.00	4.06
Th2 (CCR4+ CXCR3- CCR6-)	5.31	1.21	7.18	3.41
CD8				
CD62L+ CD44-	68.38	9.41	66.20	11.06
CD62L+ CD44+	20.30	8.01	24.02	10.13
CD62L- CD44+	4.07	1.23	3.26	0.89
CD62L- CD44-	1.15	1.28	1.51	1.60

KPC-Brca1 mice (with confirmed tumor-burden by BLI) were treated daily by oral gavage with 30 mg/kg BMS-911543 or vehicle control for 2 weeks. Splenocytes were analyzed for changes in circulating immune populations.
